# Ranzcp efforts to improve access to funded treatments

**DOI:** 10.1192/j.eurpsy.2021.1048

**Published:** 2021-08-13

**Authors:** J. Allan

**Affiliations:** President, Royal Australian and New Zealand College of Psychiatrists (RANZCP), Melbourne, Australia

**Keywords:** treatments, rTMS, Quetiapine

## Abstract

**Introduction:**

Australia has a universal health insurance scheme covering part costs for private mental health care and which supports the public system. The Medical Benefits Schedule (MBS) schedule provides a recommended fee for each service, the amount the Australian Government thinks the service should cost. Many patients still pay a gap fee for the service. Similarly a system for medications, the Pharmaceutical Benefits Scheme (PBS) subsidises the cost of medicines for most medical conditions. As new evidence emerges in the treatment of psychiatric conditions, it is important that the MBS and PBS are updated so patients receive subsidised best practice treatment.

**Objectives:**

To provide an overview of RANZCP efforts to expand treatment availability through evidence and advocacy to government.

**Methods:**

The RANZCP made submissions to the independent Medical Services Advisory Committee (MSAC) requesting an MBS listing for repetitive transcranial magnetic stimulation (rTMS) for treatment of antidepressant medication-resistant major depressive disorder. Submissions were made to the independent Pharmaceutical Benefits Advisory Committee (PBAC) to request ability to prescribe quetiapine in 25mg ranges for maintenance therapy.

**Results:**

Following RANZCP submissions, the MSAC supported public funding for initial treatment with rTMS for adults with major depression who have tried antidepressant medicine or psychological therapy and remain unwell. The PBAC has recommended changes allowing prescription of 25mg quetiapine tablets for maintenance therapy for acute mania, bipolar 1 disorder and in the treatment of schizophrenia following RANZCP submission.

**Conclusions:**

The RANZCP has achieved access to treatments to provide optimal symptom relief for people living with mental illness.

